# *Rickettsia* spp. in Finnish Ixodid ticks

**DOI:** 10.1186/s13071-025-07090-6

**Published:** 2025-11-24

**Authors:** Hanna Vauhkonen, Fathiah Zakham, Liina Voutilainen, Ronn Keinänen, Katja Lind, Thanakorn Niamsap, Petteri T. Puonti, Robert S. Castrén, Teemu Smura, Ruut Joensuu, Simo Nikkari, Elina Tonteri, Olli Vapalahti, Essi M. Korhonen, Anu Jääskeläinen, Anne J. Jääskeläinen, Tarja Sironen, Paula M. Kinnunen

**Affiliations:** 1 Department of Virology, Faculty of Medicine, University of Helsinki, Helsinki, Finland; 2 Centre for Military Medicine, Finnish Defence Forces, Helsinki, Finland; 3 Finnish Institute for Health and Welfare, Helsinki, Finland; 4 Department of Veterinary Biosciences, Faculty of Veterinary Medicine, University of Helsinki, Helsinki, Finland; 5 Department of Geosciences and Geography, University of Helsinki, Helsinki, Finland; 6 University of Helsinki and Helsinki University Hospital, HUS Diagnostic Centre, Helsinki, Finland; 7 Companion Animal Business Unit, Nordic Cluster, MSD Animal Health, Espoo, Finland

**Keywords:** Cat, Dog, Human, Infection, Ixodid tick, One Health, PCR, *Rickettsia*, Rickettsiosis, Zoonosis, Finland

## Abstract

**Background:**

*Rickettsia* spp. are intracellular, arthropod-borne bacteria pathogenic to humans and animals. The clinical manifestations of rickettsial infections range from mild to severe, but diagnosis is sometimes missed owing to mild symptoms or empirical antibiotic treatment for suspected tick-borne diseases. This study aimed to determine the prevalence of *Rickettsia* spp. in ticks across Finland, explore possible regional variations, identify the infecting *Rickettsia* species, and assess rickettsial exposure of certain human patients.

**Methods:**

We analysed 5101 ticks from 20 locations, collected by flagging, crowdsourcing, or removal from pet cats and dogs between 2003 and 2021 in three study sets. Tick species were determined by quantitative PCR (qPCR), Sanger sequencing, or morphology. Rickettsial prevalences were determined by qPCR, with species confirmed by Sanger sequencing. Indirect immunofluorescence assay was used for detecting Rickettsial antibodies in human samples.

**Results:**

*Ixodes ricinus* predominated in southern Finland, while *Ixodes persulcatus* was found in central Finland and along the western coast up to southern Lapland. Occasional *I. ricinus–persulcatus* hybrids were observed in central southern Finland where both species co-occurred. *Rickettsia* DNA was more frequently detected in ticks collected from regions dominated by *I. ricinus* compared to those dominated by *I. persulcatus*. However, variations in sampling and processing may limit the comparability of these results. Ticks collected from dogs and cats contained *Rickettsia* spp. more frequently than those collected by flagging. *Rickettsia helvetica* was the primary species identified, with sporadic *Candidatus* Rickettsia tarasevichiae found exclusively in *I. persulcatus*. Among 226 sera from patients suspected of tick-borne encephalitis, 5.8% showed IgG reactivity against *Rickettsial* antigens, but titres were below the 1:512 threshold for confirmed infection.

**Conclusions:**

This study highlights the need to better understand environmental or host-linked factors influencing *Rickettsial* prevalence and emphasizes the importance of monitoring in areas prone to tick expansion due to climate change. The findings underscore the potential for *Rickettsial* diseases, necessitating enhanced diagnostic frameworks to address disease burden and improve surveillance.

**Graphical Abstract:**

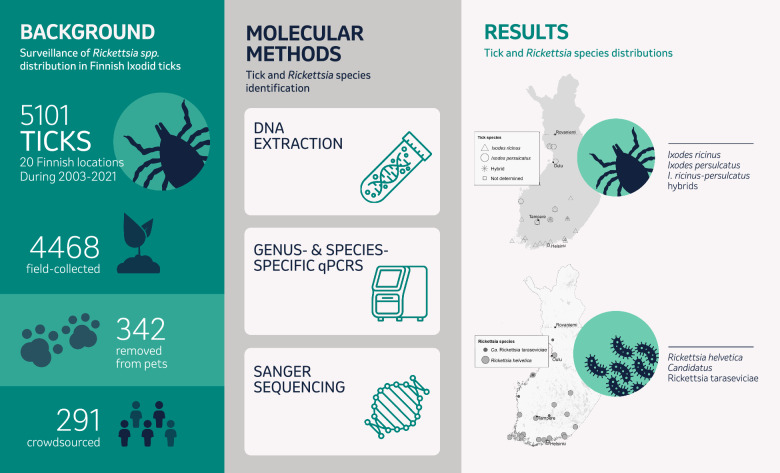

**Supplementary Information:**

The online version contains supplementary material available at 10.1186/s13071-025-07090-6.

*Rickettsia* spp. are intracellular arthropod-borne bacteria, many of which are pathogenic to humans as well as to wild and domestic animals. While certain rickettsial species are non-pathogenic arthropod endosymbionts [1, 2], a growing number of pathogenic species have been described worldwide [3]. The clinical severities of rickettsial infections vary from asymptomatic or mild, self-limiting disease to life-threatening conditions [4]. The general symptoms include fever, malaise, lymphadenopathy and rash, and, sometimes, almost pathognomonic black eschar. If diagnosed early enough, even severe cases can be treated by tetracycline antibiotics. However, misdiagnosis is possible in mild cases where patients do not seek medical care, and, as tetracycline antibiotics are potentially used when suspecting tick-borne infections, undiagnosed rickettsial infections are treated as well [5].

Traditionally, the genus *Rickettsia* has been categorized into two main groups on the basis of serological pattern, the Typhus group (TG) and the Spotted fever group (SFG), which have been named after the characteristic human disease symptoms. Scrub typhus, caused by *Orientia* spp., has been grouped occasionally under rickettsial diseases as well [6]. However, a more detailed grouping, based on whole-genome sequencing and phylogenetics, has been suggested recently, splitting SFG into two distinct groups, SFGI and SFGII, while one SFG Rickettsia, *R. helvetica*, as a separate clade sharing features with other rickettsial groups [7]. The SFGI rickettsiae and *R. helvetica* are transmitted by ticks (occasionally by mites), whereas the other pathogenic *Rickettsia* groups (TG and SFGII) are typically transmitted by arthropods other than ticks, i.e. lice, mites, and fleas [7]. TG rickettsiae are considered to relate to regions with lower hygiene and European cases have mainly been detected in returning travellers [8]. TG rickettsiae, e.g., louse-born *R. prowazekii*, are also recognized as biothreats with history and potential for deliberate use [9]. The SFGI group has been constantly expanding due to the newly described species [7].

Specific tick species serve as vectors for particular species of SFG rickettsiae, therefore, specific rickettsial diseases can be considered endemic within the distribution range of vector species [3]. However, climate change-driven expansion of tick species to new areas may result in rickettsial disease emergence in unforeseen regions [10]. The main tick species in Finland, *Ixodes ricinus* (sheep/castor bean tick) and *Ixodes persulcatus* (taiga tick), have been found to carry *R. helvetica*, *R. monacensis,* and *Candidatus* R. tarasevichiae [11]. The clinical manifestations of *R. helvetica* and *R. monacensis* infections are usually mild and patients do not need medical care, however, severe conditions cannot be excluded [12, 13]. The pathogenicity of *Ca.* R. tarasevichiae is still unclear. It has been considered an emerging pathogen as it has been associated with clinical disease in China [14].

The laboratory diagnosis of *Rickettsia* spp. infections is based on either serology or molecular (mainly PCR-based) methods [15]. Molecular methods detect the presence of DNA of the infectious agent and are mainly restricted to the acute phase, whereas serology detects the immune response in the later phases of the disease [3, 15]. Additionally, sample material plays a role. Certain rickettsial species produce eschars at the site of the arthropod bite, and the pathogen may be detected in a tissue biopsy or swab by PCR or in biopsy by immunohistochemistry. By contrast, blood samples are seldom useful, as they are rarely PCR-positive even in the acute phase, thus challenging rapid diagnostics [15]. In many Nordic countries, i.e., Sweden, Norway, and the Åland islands, Finland, *R. helvetica* seropositivity has been detected in individuals with proven tick bites [16, 17] or individuals living in tick-dense areas [18]. In Europe, *Rickettsia* spp. have been detected occasionally in clinically ill dogs, and serological testing was found to outperform molecular (PCR) methods and reveal undiagnosed infections [19, 20]. Whether or not dogs show signs of infection, they can be used as sentinels of rickettsial and other tick-borne infections [21]. Although sporadic rickettsioses have been detected in returning Finnish travellers [22], to the best of our knowledge, comprehensive data on human or animal clinical cases and seroprevalence rates are still lacking in continental Finland.

Previous studies on Finnish ticks have estimated an overall *Rickettsia* spp. prevalence of 13.9% in *I. ricinus* and 6.5% in *I. persulcatus* [11]. *Rickettsia helvetica* and sporadically *R. monacensis* have been found in both tick species and *Ca.* R. tarasevichiae in *I. persulcatus* only [11]. The *Rickettsia* spp. prevalences were reported in a country-wide manner, addressing three large collection areas (*I. ricinus*-dominated southern Finland, 12.6%; sympatric central Finland with both species present, 10.7%; and *I. persulcatus*-dominated northern Finland, 6.0%). However, studies in Estonia, which resides near Finland and shares the same tick and rickettsial species, have revealed location-specific differences in prevalences [23, 24]. For example, ticks collected from rodents in Pärnumaa were more often (19.3%) infected than ticks collected from rodents in other sites (2.6–8.1%, Saaremaa to Lääne-Virumaa, respectively) [25]. Therefore, we aimed to reveal whether similar focal differences are present in Finland.

To assess the occurrence of *Rickettsia* spp. and reveal possible focal differences in Finland, we analyzed a total of 5101 Ixodid ticks, collected from 20 locations between 2003 and 2021, in three separate study sets (Fig. 1, Table 1). Briefly, ticks in Set 1 (*n* = 3472) were collected during 2003–2015 by flagging in 12 separate locations and were mainly analysed in pools. In Set 2, ticks (*n* = 342, removed from pet cats and dogs) were obtained during 2020–2021 by ten veterinary clinics throughout Finland [26]. Ticks in Set 3 (*n* = 1155) were collected during 2017–2021 from four separate locations by flagging (*n* = 864) or crowdsourcing (*n* = 291), and in a subset of the collections, the nymphs were analyzed in pools. All ticks were nymphs or adults, except four larvae in Set 1. The pooling strategies and collection sites of questing ticks in Sets 1 and 3 were originally targeted to monitor TBEV presence. The pool sizes varied between 1 and 30 per pool depending on the prevalence of the target pathogens, the specific aims of the given sampling, and the resources available for each study. The corresponding laboratory procedures were carried out during 2015–2017, 2020–2021, and 2021–2022. Figure 1 illustrates the collection areas and approximated tick amounts included in each sample set.

**Fig. 1:**
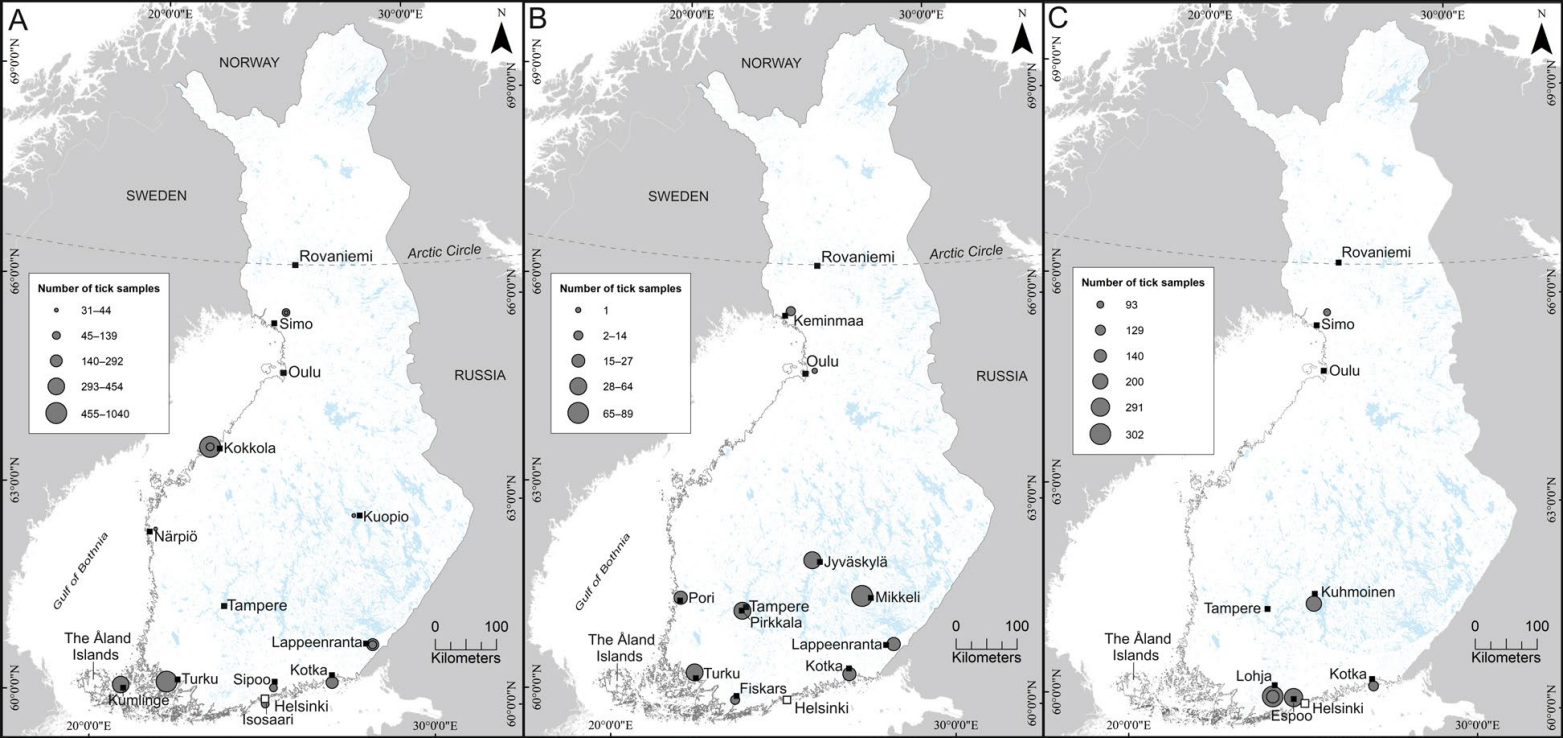
Tick collection sites in the three sample sets with approximated sample sizes. Panel A indicates the collection sites of Set 1. Panel B indicates the locations of veterinary clinics collecting ticks from pets included in Set 2. Panel C indicates the collection sites of Set 3. Maps were generated in ArcMap (version 10.8, Esri, Redlands, CA, USA)

**Table 1:** Sample collections and results for set 1, set 2, and set 3

Set 1 area, year	*N* tested (*N* pools)	Tick species^a^	*N* positive (species)	Occurrence (%) (95% CI)/Minimum occurrence (%)^b^	*N* gltA sequences^c^ (species)	Seq success (%)	Rickettsial species
Isosaari Island, 2005	96 (11)	96r/-/-	5 (r)	5.2	5 (r)	100	*R. helvetica*
Kokkola archipelago, 2003	139 (15)	Not determined	2	1.4	2	100	*R. helvetica*
Kokkola archipelago, 2004	980 (102)	-/980p/-	3 (p)	0.2	2 (p)	66.7	*Ca*. R. tarasevichiae
Kotka archipelago, 2011	194 (70)	194r/-/-	14 (r)	7.2	12 (r)	85.7	*R. helvetica*
Kumlinge Island, 2003	454 (46)	454r/-/-	2 (r)	0.5	2 (r)	100	*R. helvetica*
Kuopio, 2010	44 (5)	44r/-/-	2 (r)	4.5	2 (r)	100	*R. helvetica*
Lappeenranta, 2005	292 (29)	292r/-/-	23 (r)	7.9	16 (r)	69.6	*R. helvetica*
Lappeenranta, 2010	101 (11)	101r/-/-	7 (r)	6.9	7 (r)	100	*R. helvetica*
Närpiö, 2008	36	-/36p/-	1 (p)	2.7 (0.49–14.2)	1 (p)	100	*Ca*. R. tarasevichiae
Simo, 2009	98 (51)	-/98p/-	0	0	–	–	–
Simo, 2015	31	-/31p/-	0	0 (0.00–11.0)	–	–	–
Sipoo + Karhusaari Island, 2013	99 (48)	99r/-/-	15 (r)	15.2	13 (r)	86.7	*R. helvetica*
Turku archipelago, 2007	1040 (315)	1040r/-/-	9 (r)	0.9	9 (p)	100	*R. helvetica*
Total Set 1	3604	2320r/1145p	83 (77r/4p)	2.3	71 (68r/1p)	86.6	

Set 2 Vet practice location, year(s)	*N* tested	Tick species	*N* positive (species)	Occurrence (%) (95% CI)	*N* gltA sequences (species)	Seq success (%)	Rickettsial species
Fiskars, 2020–2021	14	14r/-/-	2 (r)	14.3 (4.0–39.9)	0	0	–
Jyväskylä, 2020–2021	44	38r/5p/-^d^	29 (24r/5p)	65.9 (51.1–78.1)	16 (15r/1p)	66.7	*R. helvetica*
Keminmaa, 2021	11	1r/10p/-	0	0 (0.0–25.9)	–	–	–
Kotka, 2020	25	25r/-/-	20 (r)	80.0 (60.9–91.1)	8 (r)	40.0	*R. helvetica*
Mikkeli, 2020–2021	89	88r/-/1 h	43 (r)	48.3 (38.2–58.6)	27 (r)	62.8	*R. helvetica*
Lappeenranta, 2020	23	23r/-/-	11 (r)	47.8 (29.2–67.04)	6 (r)	54.5	*R. helvetica*
Oulu, 2020	1	1r/-/-	1 (r)	^e^	1 (r)	100.0	*R. helvetica*
Pirkkala, 2020–2021	64	29r/35p/-	14 (11r/3p)	21.9 (13.5–33.4)	7 (5r/2p)	50.0	*R. helvetica*
Pori, 2020–2021	27	27r/-/-	17 (r)	63.0 (44.23–78.5)	15 (r)	88.2	*R. helvetica*
Turku, 2020–2021	44	44/-/-	3 (r)	6.8 (2.4–18.2)	0	0	
Total Set 2	342	289r/51p/1 h	140 (132r/8p)	40.9 (35.9–46.2)	80 (77r/3p)	58.8	

Set 3 area, year	*N* tested (*N* pools)	Tick species	*N* positive (species)	Occurrence (%) (95% CI)/minimum occurrence (%)	*N* gltA sequences (species)	Seq success (%)	Rickettsial species
Espoo, 2017^f^	291	291r/-/-	16 (r)	5.5 (3.4–8.7)	8 (r)	50.0	*R. helvetica*
Kotka Kuutsalo Island, 2018	129 (102)	128r/-/1 h	17 (r)	13.2	9 (r)	75.0	*R. helvetica*
Kuhmoinen, 2020	200	18r/173p/9 h	3 (1r/2p)	1.5 (0.5–4.3)	2 (p)	66.7	*Ca*. R. tarasevichiae
Lohja, 2019	140 (64)	140r/-/-	17 (r)	12.1	12 (r)	70.6	*R. helvetica*
Lohja, 2021	302 (64)	302r/-/-	22 (r)	7.3	22 (r)	100	*R. helvetica*
Simo, 2020	93	-/93p/-	1 (p)	1.1 (0.2–5.8)	1 (p)	100	*Ca.* R. tarasevichiae
Total Set 3	1155	879r/266p/10 h	76 (73r/3p)	6.6	54 (51r/3p)	71.1	
Total Sets 1–3	5101		299 (282r/15p)	5.8	205 (196r/7p)	68.8	

Tick collection sites, number of samples (and pool numbers in Sets 1 and 3) tested, Ixodes species, number and tick species of Rickettsia spp. –positive samples, occurrence with 95% confidence intervals, number of obtained gltA. sequences, sequencing success, and rickettsial species obtained by sequencing

^a^Tick species: r = *I. ricinus*, p = *I. persulcatus*, h = *I. ricinus-persulcatus* hybrid, species determination methods listed in Additional File Table S1

^b^Minimum prevalence: assuming only one sample in a pool is positive

^c^N *gltA* sequences; number of *gltA* (citrate synthase gene) sequences obtained by sequencing

^d^One Jyväskylä tick identified as *Rhiphicephalus sanguineus*, *Rickettsia* spp. negative

^e^Tick number too low for reliable calculation

^f^Sample set obtained by crowdsourcing

Slightly different molecular methods were used for tick species identification, rickettsial occurrence, and species determination in each set. Briefly, DNA was extracted from tick homogenates by commercial kits: either TriPure Reagent DNA method, AllPrep DNA/RNA kit (Qiagen, Hilden, Germany), GenJET Genomic DNA purification kit (Thermo Fisher Scientific, Waltham, MA, USA), or DNeasy Blood and Tissue kit (Qiagen). Tick species was determined by either morphology or analysis of internal transcribed spacer 2 (*ITS2*) gene or mitochondrial 16S RNA gene. The presence of rickettsial DNA and the corresponding species were determined by quantitative genus-specific PCR and Sanger sequencing of citrate synthase (*gltA*) gene, respectively. Detailed sampling information, life stages, pool sizes, DNA extraction methods and the molecular methods in tick species and *Rickettsia* analyses are described in Additional File 1: Text S1, Tables S1, and S2. Subsets of the samples have been previously screened for other pathogens [26–32], described in Additional File 1: Table S1.

We found *I. ricinus* to prevail in southern Finland and *I. persulcatus* in northern Finland, and sympatric occurrence of both species in the central part of southern Finland: in Pirkkala and Jyväskylä (Set 2, previously reported by Zakham et al. [26]), and in Kuhmoinen (Set 3). Table 1 provides a detailed description and Fig. 2 a compilation of the results of this study. Our species distribution data are in line with previous reports by Laaksonen et al. [11] and are supported also by habitat suitability predictions by Uusitalo et al. [33]. Intriguingly, 4.5% of the field-collected ticks from Kuhmoinen (Set 3) were identified as *I. ricinus*–*persulcatus* first-generation hybrids using duplex real-time quantitative PCR [34], modified by Zakham et al. [32]. Respective hybrids have previously been described in regions co-habited by the two species, e.g., Estonia [35], Lempäälä, and Tampere [26, 36]. One hybrid was found in Kotka (Set 2), and one *Rhipicephalus sanguineus* in Jyväskylä (Set 2). The *R. sanguineus* was collected from a traveling dog returning from Spain [26]. The tick was not infected with *Rickettsia*.

**Fig. 2:**
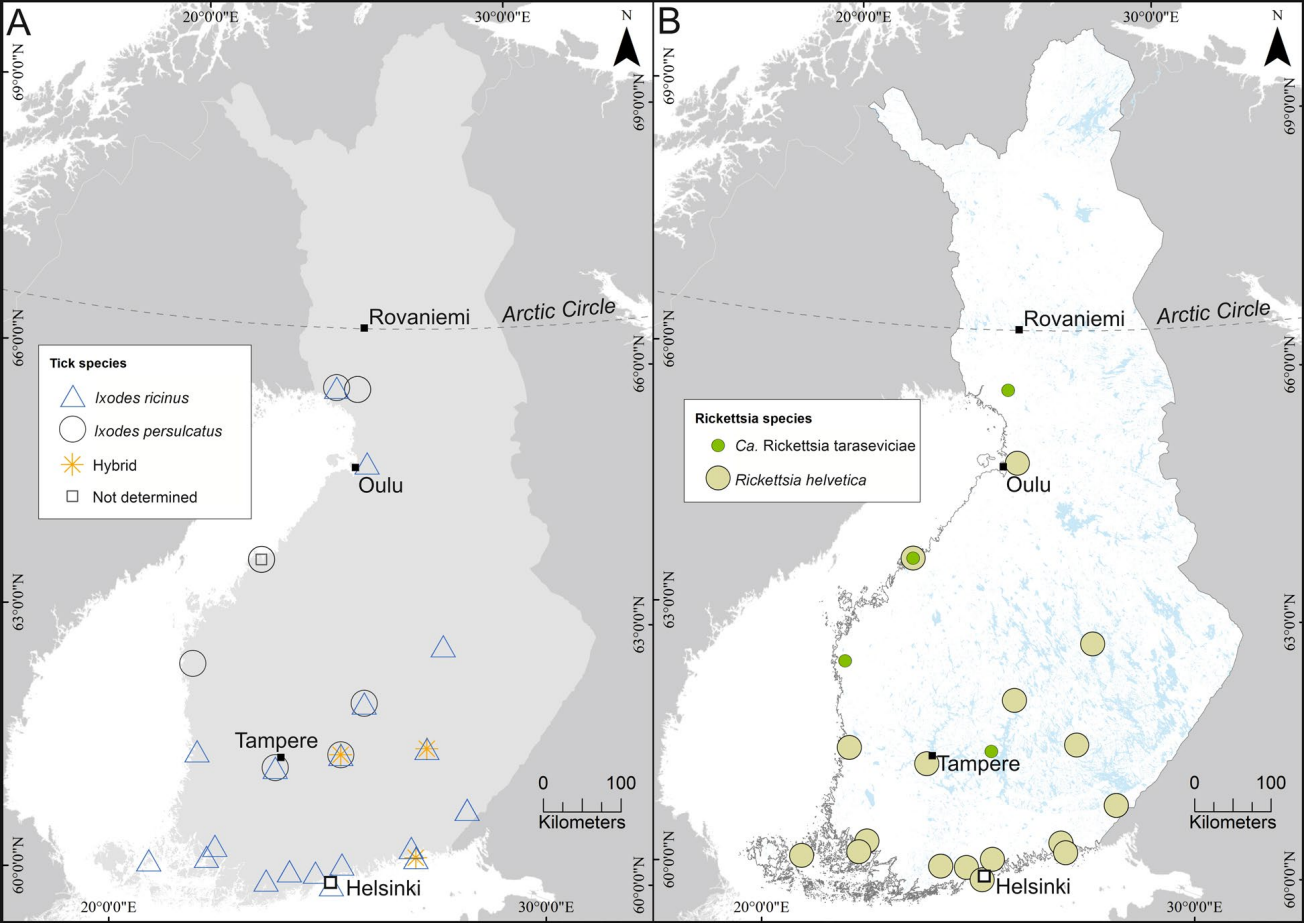
Presence of tick and Rickettsia spp. in the collection sites. Panel A shows the presence of *Ixodes ricinus* (red), *Ixodes persulcatus* (blue) and *I. ricinus-persulcatus* hybrids (pink) in the collection sites. Collection sites where tick species detection was based on methods other than molecular methods are indicated by a small grey box symbol. Panel B shows *Rickettsia* species (*R. helvetica* light green, *Ca.* R. tarasevichiae, dark green) in the respective collection sites. Maps were generated in ArcMap (version 10.8, Esri, Redlands, CA, USA)

Overall, in Sets 2 and 3, *Rickettsia* spp. infections were more common in *I. ricinus* (132/289, 45.7% and 73/879, 8.3%, respectively) than in *I. persulcatus* (8/51, 15.7% and 3/266, 1.1%) (*p* < 0.001, 2-sample *z*-test). The 6.6% overall occurrence obtained from Set 3 is in line with previous observations [11], whereas the considerably higher (40.9%) occurrence in ticks collected from pets (Set 2) was unexpected and could not be explained by differences in laboratory procedures or sample origin. Indeed, the crowdsourced samples used by Laaksonen et al. [11] were mainly obtained from companion animals during 2015 and probably are geographically biased toward residential areas [37]. However, while the specific locations of ticks collected from pets cannot be verified because of scant traveling details provided by owners, crowdsourcing is still considered a valid method of monitoring pathogen occurrences in larger areas [11, 37]. In total, 27 of the veterinary clinic pets included in our study had more than one tick, and only 3 pets had two engorged rickettsia-positive ticks, suggesting a minor role for infection by co-feeding. Since most of the samples in Set 1 were analyzed in pools of varying sizes between and within collection sites, occurrence could be approximated only to the lowest percentage i.e., assuming only one positive sample in the pool, resulting in a total minimum occurrence of 2.3% (83/3604).

At locations where the only tick species found was *I. ricinus*, the rickettsial occurrences varied within and between sample sets (0–80.0%), with Set 2 having the highest occurrences. Southwestern Finland and its archipelago had the lowest occurrences (0.5–6.8%). Accordingly, Southwestern Finland and the Åland islands have previously been described to present lower rickettsial occurrences (0–5.1%) than the national average (10.8%) [16, 34]. At locations where the only tick species found was *I. persulcatus,* the occurrences were lower (0–2.7%), as also reported previously [11]. The only tick sample from Oulu, Northern Finland (Set 2), was *Rickettsia* spp*.* –positive *I. ricinus* detached from a dog and was not considered to represent the whole area, as it could have originated from a migratory bird or a traveling dog.

Among the three sympatric locations, Kuhmoinen was dominated by *I. persulcatus* (173/200, 86.5%), whereas in Pirkkala *I. persulcatus* was nearly equally abundant (35/64, 54.7%) as *I. ricinus.* In Jyväskylä, *I. persulcatus* was present only as a minority (5/44, 11.4%). The overall rickettsial occurrence was 1.5% in Kuhmoinen (Set 3, two *I. persulcatus* and one *I. ricinus*). In Jyväskylä and Pirkkala (Set 2), overall *Rickettsia* spp. occurrences of 65.9% (29/44) and 21.9% (14/64) were observed, respectively. All (5/5) *I. persulcatus* ticks and 63.2% (24/38) of *I. ricinus* in Jyväskylä and 8.6% (3/35) of *I. persulcatus* and 37.9% (11/29) *I. ricinus* in Pirkkala were *Rickettsia* spp. positive. The nine *I. ricinus—persulcatus* hybrids of Kuhmoinen and the one of Kotka were *Rickettsia-*negative.

The main rickettsial species observed by sequencing was *R. helvetica,* and it was most often found in *I. ricinus*-dominated regions. Six *Ca.* R. tarasevichiae-positive samples or pools were found in *I. persulcatus* ticks (Sets 1 and 3) in Kuhmoinen, Simo, Närpiö, and Kokkola 2004. Only *R. helvetica* was found in Set 2, including the five sequenceable *I. persulcatus* samples in Jyväskylä and Pirkkala. No *R. monacensis* or any other rickettsial species were found in the entire study. The sequencing success rate ranged from 40 to 100% between the sample sets, Set 2 with the lowest and Set 3 with the highest success rate (Table 1). Variation in DNA extraction methods and *gltA* sequencing strategies (un-nested *versus* nested) between the study sets may explain the differences in success rates. Although the nested PCR strategy in Sanger sequencing has been suggested as more sensitive, the better sequencing success observed in Set 1 with an un-nested strategy could be explained by pooled samples. In addition, samples with qPCR *C*_t_ values above 35 failed more often to produce sequence, reflecting either lower rickettsial proportion or lower DNA quality of the sample. Previously, Laaksonen et al. [11] reported a sequencing success rate of 68.1%.

We found no obvious reason for the substantially higher occurrences in our collection of ticks detached from pets (Set 2). Six engorged rickettsia-positive ticks were collected from three pets, two ticks from each, suggesting the possibility of infection by co-feeding or acquiring rickettsial infection from the host. However, different collection years or seasonal variation affecting the habitat might explain the differences [37–39]. As different nutritional or climatic factors have been suggested to affect the tick microbiome [40, 41], rickettsial abundances within the tick host may also depend on environmental conditions, including weather. In addition, competition between pathogenic and nonpathogenic *Rickettsia* species has been observed within the vector cell, e.g., a nonpathogenic endosymbiont may hinder the infection of pathogenic species [42], therefore explaining patchy occurrences. For more accurate comparison of the occurrence of pathogens in ticks collected from pets with those collected from the environment, environmental sampling close to the veterinary clinics collecting ticks from pets and around the same time is needed. This will be considered in future studies. To our knowledge, no seroprevalence data on *Rickettsia* spp. in companion animals living in Finland exist, and thus, their actual exposure is unknown. Antibodies showing high exposure to SFG rickettsiae have been reported from Sweden and Germany [43, 44].

To estimate the prevalence of human rickettsial infections in Finland, sera from 226 individual patients suspected of tick-borne encephalitis (TBE) were obtained from late May to early July 2018 throughout the country. The samples were screened for rickettsial antibodies using commercial immunofluorescence assay (IFA) to detect possible undiagnosed rickettsioses. Sample and IFA analysis details are provided in Additional File 2: Text S2. Overall, 13 sera (5.8%) were considered borderline or low positive, and these might be due to an unspecific reaction. There were no high positive findings using commercial IFA, and no infection caused by *Rickettsia* spp. was detected (Additional File 1: Table S3). As no data on potential eschars or traveling and no paired sample sera were available for borderline positive patients, no conclusions could be made regarding endemic, travel-related, or past or present infections. For comparison, in Denmark, 2147 individuals were tested for rickettsial antibodies during 2008–2015, yielding 561 positives (26%; cut-off 1:512), 86% of which were SFG-positive [45].

In conclusion, we describe *Rickettsia* spp. occurrence in 20 geographical locations in Finland Ticks collected from pets appeared to be more frequently *Rickettsia*-positive than those collected by other methods, and *I*. *ricinus* ticks seem to be more commonly infected with rickettsiae than *I. persulcatus* ticks. The reason for regional differences in rickettsial occurrence—whether attributable to the predominance of specific tick species, climatic or nutritional factors, interactions with competing endosymbiotic organisms, or other yet unidentified determinants—remains to be elucidated. The variable sampling strategies presented in this study limit the assessment of these determinants. We found no clear rickettsial seropositivity in patients suspected of TBE; however, for verified serology-based diagnosis, paired serum samples showing that rising titres are needed. In any case, our findings underscore the common occurrence of *Rickettsia* spp*.* in ticks, and hence, the potential for rickettsial diseases, necessitating enhanced diagnostic frameworks to address disease burden and improve disease surveillance. Since potentially pathogenic rickettsial species clearly exist in Ixodid ticks in Finland, rickettsioses need to be included among differential diagnoses by both physicians and veterinarians.

## Supplementary Information

Additional file 1.

Additional file 2.

## Data Availability

The *Rickettsia* sequences obtained in the study were identical to GenBank accessions U59723.1 (*R. helvetica*) or AF503167.2 (*Ca.* R. tarasevichiae). The datasets are available from the corresponding author upon request.
