# Supplementary material for: Rickettsia spp. in Finnish Ixodid ticks
Source: Parasit Vectors. 2025 Nov 24;18:485. doi: 10.1186/s13071-025-07090-6 (PMC12642282; doi:10.1186/s13071-025-07090-6)
Supplement: Supplementary file 1 — Additional file 1. [file 13071_2025_7090_MOESM1_ESM.docx]

**Additional file 1**

**Text S1**

**Tick collection, DNA extraction and molecular analyses**

Ticks were collected either by cloth dragging (flagging; Sets 1 and 3), crowdsourcing (Set 3 Espoo area) or tick detachment at veterinary clinics [26] (Set 2). Ticks were homogenized in either TriPure Isolation Reagent (Roche Applied Sciences, Basel, Switzerland) or Dulbecco’s PBS with 0.2% BSA and using TissueLyzer II at 30 rps. DNA was extracted from the homogenates either using the TriPure Reagent DNA method, AllPrep DNA/RNA kit (Qiagen, Hilden, Germany), GenJET Genomic DNA purification kit (Thermo Scientific) or DNeasy Blood and Tissue kit (Qiagen). In Set 1, a varying number of ticks were pooled at some collection sites before homogenization. DNA extraction methods used for each subset as well as previous reports on different pathogens are clarified in Table S1.

The veterinarians / veterinary nurses at the selected clinics (in Mikkeli, Lappeenranta, Pori, Jyväskylä, Keminmaa, Fiskars, Pirkkala, Kotka, Oulu, and Turku) searched for ticks in dogs and cats, collected them with SafeCards (one per animal) and transferred with tweezers (avoiding squeezing the ticks; preferably from legs) to sample tubes filled with RNALater, and filled in a data sheet. Ticks from one animal were put to the same tube. Ticks were stored up to a couple of months at the clinics until transported or sent to the laboratory.

Tick species were determined either by morphology, dual-labelled real-time quantitative PCR (RT-qPCR) of the internal transcribed spacer 2 (*ITS2*) gene [34, modified by Zakham et al. [32]], amplification of *ITS2* [46] or mitochondrial 16S RNA gene [47] followed by Sanger sequencing, or, in a vast minority of subsets belonging to Set 1, by assumption of the prevailing species. Sanger sequencing was performed in the Finnish Institute of Molecular Medicine (FIMM).

*Rickettsia spp*. infection was determined (either sample-wise or pool-wise) by generic RT-qPCR addressing the citrate synthase (*gltA*) gene [48, 49]. Positive samples/pools were amplified to produce longer *gltA* gene fragments, which were subsequently Sanger sequenced in FIMM. A nested PCR method [50] was used in Sets 2 and 3, whereas the sequencing of Set 1 was based on a single PCR reaction [51]. The sensitivity of the generic qPCR method used in Sets 1 and 2 was validated using serial dilutions of *R. helvetica*-infected cell culture DNA (courtesy of Martin Pfeffer). TaqMan™ Exogenous Internal Positive Control Reagent (Thermo Fisher Scientific, Vantaa, Finland) was employed to assess the presence of potential inhibitors in Set 1. Primer sequences and respective amplicon sizes are available in Table S2. The rickettsial species were identified by using the BLAST program available at NCBI.

Statistical significances and 95% confidence intervals were calculated from the obtained proportions and sample numbers using EpiTools epidemiological calculators (<https://epitools.ausvet.com.au/ciproportion>) and the Wilson method suggested for small sample sizes.

**Supplementary references, if not cited in main text**

46 Rumer L, Sheshukova O, Dautel H, Mantke OD, Niedrig M. Differentiation of medically important euro-asian tick species *Ixodes ricinus*, *Ixodes persulcatus*, *Ixodes hexagonus*, and *Dermacentor reticulatus* by polymerase chain reaction. Vector Borne Zoonotic Dis. 2011 Jul;11(7):899–905. doi:10.1089/vbz.2009.0191

47 Caporale DA, Rich SM, Spielman A, Telford SR, Kocher TD. Discriminating between Ixodes ticks by means of mitochondrial DNA sequences. Mol Phylogenet Evol. 1995 Dec;4(4):361–5. doi:10.1006/mpev.1995.1033

48 Stenos J, Graves SR, Unsworth NB. A highly sensitive and specific real-time PCR assay for the detection of spotted fever and typhus group Rickettsiae. Am J Trop Med Hyg. 2005 Dec;73(6):1083–5. doi:10.4269/ajtmh.2005.73.1083

49 Labruna MB, Whitworth T, Horta MC, Bouyer DH, McBride JW, Pinter A, et al. *Rickettsia* species infecting *Amblyomma cooperi* ticks from an area in the state of são Paulo, Brazil, where Brazilian spotted fever is endemic. J Clin Microbiol. 2004 Jan;42(1):90–8. doi:10.1128/jcm.42.1.90-98.2004

50 Igolkina Y, Bondarenko E, Rar V, Epikhina T, Vysochina N, Pukhovskaya N, et al. Genetic variability of *Rickettsia* spp. in *Ixodes persulcatus* ticks from continental and island areas of the Russian Far East. Ticks Tick Borne Dis. 2016 Oct;7(6):1284–9. doi:10.1016/j.ttbdis.2016.06.005

51 Roux V, Rydkina E, Ermeeva M, Raoult D. Citrate synthase gene comparison, a new tool for phylogenetic analysis, and its application for the rickettsiae. Int J Syst Bacteriol. 1997 Apr 1;47(2):252–61. doi:10.1099/00207713-47-2-252

**Table S1.** Sample sets, DNA extraction, tick species determination methods and references for samples used in previous studies addressing different pathogens.

| Set | Location, year | N ticks | N available life stages (F/M/N/L)^a^ | N pools (pool size) | DNA extraction method | Tick species determination method **^b^** | Previous pathogen studies on the same sample set |
| --- | --- | --- | --- | --- | --- | --- | --- |
| 1 | Isosaari Island 2005 | 96 | 16/26/54/- | 11 (1-10)^c^ | TriPure | Morphology | [28] |
|  | Kokkola archipelago 2003 | 139 | -/1/138/- | 15 (1-10)^c^ | TriPure | Nd |  |
|  | Kokkola archipelago 2004 | 980 | 456/453/71/- | 102(3-14) | TriPure | Morphology | [27] |
|  | Kotka archipelago 2011 | 194 | 37/74/83/- | 70 (1-30) | TriPure | *ITS2* gene sequencing | [30] |
|  | Kumlinge island 2003 | 454 | 44/23/387/- | 4 (2-14) | TriPure | Morphology | [28] |
|  | Kuopio 2010 | 44 | 18/25/1/- | 5 (3-11) | TriPure | Morphology |  |
|  | Lappeenranta 2005 | 292 | 140/138/14/- | 29 (7-10) | TriPure | Morphology | [28] |
|  | Lappeenranta 2010 | 101 | 56/45/-/- | 11 (3-11) | TriPure | Morphology |  |
|  | Närpiö 2008 | 36 | 22/13/-/- | Nd^d^ | TriPure | mt16S RNA gene sequencing | [28] |
|  | Simo 2009 | 98 | 43/55/-/- | 51 (1-3) | TriPure | mt16S RNA gene sequencing | [29] |
|  | Simo 2015 | 31 | 18/9/4/- | Nd | TriPure | Morphology |  |
|  | Sipoo + Karhusaari Island 2013 | 99 | 40/35/24/- | 48 (1-3) | AllPrep DNA/RNA | Morphology | [31] |
|  | Turku Archipelago 2007 | 1040 | 98/88/856/4 | 315 (1-30) | TriPure | mt16S RNA gene sequencing | [28] |
| 2 | Collected by ten Finnish veterinary clinics 2020-2021 **^e^** | 342 | 157/46/3/- | Nd | GeneJET, DNeasy Blood and Tissue kit (Qiagen) | dual RT-qPCR, *ITS2* gene sequencing | [26] |
| 3 | Espoo 2017 | 291 | 103/85/88/- | Nd | TriPure | dual RT-qPCR | [31, 32] |
|  | Kotka Kuutsalo Island 2018 | 129 | 38/56/35/- | 102 (1-5)^c^ | GeneJET | dual RT-qPCR |  |
|  | Lohja 2019 | 140 | 10/35/95/- | 64 (1-5)^c^ | GeneJET | dual RT-qPCR |  |
|  | Lohja 2021 | 302 | -/1/301/- | 64 (1-7)^c^ | DNeasy Blood and Tissue kit (Qiagen) | dual RT-qPCR |  |
|  | Kuhmoinen 2020 | 200 | 89/111/-/- | Nd | GeneJET | dual RT-qPCR |  |
|  | Simo 2020 | 93 | ^f^ | Nd | GeneJET | dual RT-qPCR |  |
|  | Total | 5101 |  |  |  |  |  |

^a^Life stages: F = female, M = male, N = nymph, L = larva, not available for all samples

**^b^**Primers and references for the molecular methods are available in Table S2

**^c^**Only nymphs pooled

**^d^**Nd= pooling not done

^e^Number of samples provided by each clinic available in Table 1 of main text

^f^93 adults (sexes uncertain), 1 nymph

**Table S2**. Primers used in the analysis of different sample sets of the study. Target gene names, primer sequences, amplicon sizes, references, and respective sample sets

| **Primer** | **Target gene** | **Sequence 5’>3’** | **Product size bp** | **Reference** | **Samples** |
| --- | --- | --- | --- | --- | --- |
| 16Sa | *mt16S RNA (Ixodes* spp.) | CGCCTGTTTATCAAAAACAT | 339 | [47] | Set 1 |
| 16Sb |  | CTCCGGTTTGAACTCAGATC |  |  |  |
| IXO-I2-F4 | *ITS2 (Ixodes* spp.) | TCTCGTGGCGTTGATTTGC | 64 | [34, 32] | Set 2, Set 3 |
| IXO-I2-R4 |  | CTGACGGAAGGCTACGACG |  |  |  |
| Ipe-I2-P4 | *I. persulcatus-*specific | [FAM]-TGCGTGGAAAGAAAACGAG-[BHQ1] |  |  |  |
| Iri-I2-P4 | *I. ricinus-specific* | [HEX]-TGCTCGAAGGAGAGAACGA-[BHQ1] |  |  |  |
| dITS29 | *ITS2 (Ixodes* spp.) | CCTTCCCGTGGCTTCGTCTGT | 778 | [46]**^a^** | Set 2 |
| rITS800 |  | GGGGGTTGTCTCGCCTGATGT |  |  |  |
| CS-F | *gltA* (*Rickettsia* spp.) | TCGCAAATGTTCACGGTACTTT | 74 | [48] | Set 1, Set 2 |
| CS-R |  | TCGTGCATTTCTTTCCATTGTG |  |  |  |
| CS-P |  | [FAM]-TGCAATAGCAAGAACCGTAGGCTGGATG-[BHQ1] |  |  |  |
| Rspp-F | *gltA* (*Rickettsia* spp.) | GAG AGA AAA TTA TAT CCA AAT GTT GAT | 147 | [49, 34] | Set 3 |
| Rspp-R |  | AGG GTC TTC GTG CAT TTC TT |  |  |  |
| Rspp-P |  | [Cy5]-CAT TGT GCC ATC CAG CCT ACG GT-[BHQ3] |  |  |  |
| CS535d (F) | *gltA* (*Rickettsia* spp.) | GCA ATG TCT TAT AAA TAT TC | 643 | [51]**^a^** | Set 1 |
| Rp1258 (R) |  | ATT GCA AAA AGT ACA GTG AAC |  |  |  |
| glt1 | *gltA* (*Rickettsia* spp.) | GATTGCTTTACTTACGACCC | 1087 | [50]**^a^** | Set 2, Set 3 |
| glt2 |  | TGCATTTCTTTCCATTGTGC |  |  |  |
| glt3 | *gltA* (nested) | TATAGACGGTGATAAAGGAATC | 667 |  |  |
| glt4 |  | CAGAACTACCGATTTCTTTAAGC |  |  |  |

**^a^**Method for PCR product used in Sanger sequencing.
