# Supplementary material for: Rickettsia spp. in Finnish Ixodid ticks
Source: Parasit Vectors. 2025 Nov 24;18:485. doi: 10.1186/s13071-025-07090-6 (PMC12642282; doi:10.1186/s13071-025-07090-6)
Supplement: Supplementary file 2 — Additional file 2. [file 13071_2025_7090_MOESM2_ESM.docx]

**Additional file 2**

**Text S2: Patient sera and immunofluorescence analysis (IFA)**

Blood samples (n = 226) sent to Helsinki University Hospital laboratory (HUS Diagnostic center, Helsinki, Finland) were retrieved from late May to early July 2018. The patients were seeking medical care with variable neurologic symptoms and suspicion of tick-borne encephalitis (TBE). Data about potential eschars was not available. The patients were fully anonymized for the present study, date of the possible tick-bite (or bite of another arthropod) and travel data were unavailable. Rickettsial antibodies were screened using Rickettsia IgG Immunofluorescence analysis (IFA) (IF0100G, Focus Diagnostics, CA, USA). Patient ages ranged from 2 to 89 years with a median of 48 years. Most samples came from the Helsinki and Uusimaa healthcare district (n=108), Nordlab (n=57), Åland (n=17) and Northern Savo (n=9), located in south, north, southwest, and middle of Finland, respectively. Single samples came from North Karelia, Central Finland (FIMLAB), Kymenlaakso, Päijänne-Tavastia and Southern Savo. Titers >1:512 were considered positive, <1:32 negative, and 1:32-1:512 borderline.

**Table S3**. Titers of seropositive patient sera for either Typhus group (TG titer) or Spotted fever group (SFG titer) Rickettsia. Titers show the reciprocal of the highest serum dilution producing positive reaction.

| sample # | TG titer | SFG titer |
| --- | --- | --- |
| 1 | 64 | <32 |
| 2 | 128 | 128 |
| 3 | 32 | 64 |
| 4 | 32 | 64 |
| 5 | 32 | 32 |
| 6 | 32 | 32 |
| 7 | 32 | 32 |
| 8 | 128 | 128 |
| 9 | 128 | 32 |
| 10 | <32 | 64 |
| 11 | 128 | 32 |
| 12 | 64 | 128 |
| 13 | 64 | <32 |
